# ATTACHING MORE MEANING TO THE HOME SUPPORTS ACTIVE AGING DESPITE FUNCTIONAL DECLINE

**DOI:** 10.1093/geroni/igae098.0282

**Published:** 2024-12-31

**Authors:** Björn Slaug, Marianne Granbom, Jonas Björk, Taina Rantanen, Steven Schmidt, Susanne Iwarsson

**Affiliations:** Lund University, Lund, Skane Lan, Sweden; Centre of Ageing and Supportive Environments (CASE), Lund University, Skane Lan, Sweden; Lund University, Lund, Skane Lan, Sweden; University of Jyvaskyla, Jyvaskyla, Keski-Suomi, Finland; Lund University, Lund, Skane Lan, Sweden; Lund University, Lund, Skane Lan, Sweden

## Abstract

When functional limitations increase with age, perceived qualities of the home may influence the level of activity among older adults. Active aging is the process through which people strive to maintain wellbeing when growing old. The aim of this study was to investigate whether perceived housing moderates the relationship between functional limitations and active aging. Cross-sectional data from a sub-sample (N=820; mean age=69.7; 54% women) of the Prospective RELOC-AGE was used. Functional limitations were captured by ten dichotomous questions. Active aging was assessed with the University of Jyvaskyla Active Aging Scale (UJACAS; 17 items, self-rated for four perspectives). Perceived housing was self-rated with four usability questions and the meaning of home (MOH; 28 items) questionnaire. Associations and interactions were analysed using linear regression models, adjusting for gender and educational level. Each functional limitation decreased the active aging score by almost five points (p<0.001). Usability did not moderate the relationship while MOH significantly attenuated the association between functional limitations and active aging (p=0.039). Those with high MOH had two points less decrease in active aging score compared to those with low MOH. Having a home with more personal meaning attached to it seems to provide more ability and opportunity for meaningful activities, thus supporting active aging despite functional limitations. This sheds new light on the known association between MOH and different aspects of wellbeing in old age and has relevance for further theory development.

